# Value chain analysis and sanitary risks of the camel milk system supplying Nairobi city, Kenya

**DOI:** 10.1016/j.prevetmed.2018.09.010

**Published:** 2018-11-01

**Authors:** Dishon Muloi, Pablo Alarcon, Jackson Ombui, Kisa J. Ngeiywa, Bulle Abdullahi, Patrick Muinde, Maurice K. Karani, Jonathan Rushton, Eric M. Fèvre

**Affiliations:** a University of Nairobi, Nairobi, Kenya; b International Livestock Research Institute, Nairobi, Kenya; c Centre for Immunity, Infection and Evolution, University of Edinburgh, Edinburgh, United Kingdom; d Royal Veterinary College, Hawkshead Lane, Hatfield, United Kingdom; e Leverhulme Center for Integrative Research in Agriculture and Health, London, United Kingdom; f Institute of Infection and Global Health, University of Liverpool, Liverpool, United Kingdom; g Kenya Camel Association, Nairobi, Kenya

**Keywords:** Value chain, Nairobi, Camel milk, Supply system, Governance and food safety, Dairy, Kenya

## Abstract

The camel milk trade in Kenya has evolved significantly from a small-scale business undertaken in local villages to its current status involving a large number of different stakeholders supplying urban towns, particularly Nairobi City. Despite the evident growth pattern, the supply of camel milk to Nairobi has largely remained informal, with minimal enforcement of regulations. The aim of this study was to characterise the camel milk system supplying Nairobi and assess its governance, main challenges and the potential food safety risk practices.

A value chain analysis framework was used to carry out data collection between August 2014 and July 2015. Qualitative and quantitative data were collected through focus group discussions and key informant interviews with stakeholders operating in different nodes of the value chains.

Three milk value chains supplying Nairobi were identified and mapped: the Isiolo chain, the Kajiado chain and the camel milk processing company chain. Overall, the results indicate that 94% of the milk supplied to Nairobi city is informally traded (traded without any effective regulation), while 6% originates from a formal milk processing company. In the informal chains, milk traders (mostly women) were reported to play a pivotal role in the organisation and daily functioning of the chains. The processing company had partly integrated activities and reported exporting 5% of their products to regional and international markets.

Food safety themes identified were associated with i) lack of cold chain, ii) gaps in hygiene practices, particularly at farm and market levels, iii) consumption of raw camel milk, and iv) lack of food safety training, among other issues. Low level involvement by government agencies in enforcing stipulated food safety measures were reported in the informal chains, as these concentrate efforts in the regulation of dairy milk chains. Isiolo milk traders were identified as the dominant group, setting milk prices and providing sanctions.

The framework and findings obtained can help future research and policy makers to reach informed decision about what to regulate, where to target and importantly how to make the camel milk value chain more efficient and safer.

## 1 Introduction

Recent estimates suggest that more than 60% of the world’s dromedary camel population is in the four East African countries: Kenya, Somalia, Sudan and Ethiopia. Kenya is the second highest producer of camel milk in the world with an approximated production of 0.94 million litres per annum (FAOSTAT, 2014), with a value projection of more than US$ 34 million (Musinga et al., 2008). Camels’ unique adaptability to arid and marginalised areas (Schwartz, 1992; Khan et al., 2003) results in their milk constituting a significant proportion of the total diet intake for camel owning pastoral communities in Eastern Africa.

The camel milk trade in Kenya has evolved significantly from a small scale business undertaken in few local villages to the current trade involving many stakeholders in different parts of the country, and the larger East African region (Anderson et al., 2012). Despite the evident growth pattern, the subsector has largely remained informal, with minimal regulation from relevant authorities. As a result, access to markets has been challenging, with only 12% of the total milk produced marketed: 10% sold to rural consumers, and only 2% to urban markets. The remaining 88% is consumed in local households, with a significant proportion going to waste due to post harvest losses (Akweya et al., 2012). Nonetheless, with increased population growth and rural-urban migration, demand for camel milk in Nairobi has risen over the last decade (Matofari et al., 2007). Additionally, the perceived medicinal properties and associated health benefits of camel milk have acted as strong marketing tools for the product, both in Kenya and elsewhere. However, given the high level of the informal milk trade, public health issues such as the risk of milk borne zoonotic diseases are of concern (Lore et al., 2005). As such, it is crucial to understand how the camel milk value chain operates in order to assess the potential economic and food safety risks that may occur; as well as exploring how these chains can be governed, promoted and improved to make them both more successful and safer.

Despite the apparent growth and predictions of greater performance, there is little knowledge about the organisation of the camel milk value chains supplying Nairobi. A deeper understanding of the functioning of the milk supply system within the city is vital to help in designing of food safety policies. Value chain analysis (VCA) is a valuable framework for understanding temporal and spatial connectivity of people and food products and their interactions (Alarcon et al., 2017). Also, VCA provides an important framework for the identification and understanding of chain governance, challenges and structural deficiencies (Kaplinsky and Morris, 2001; Alarcon et al., 2017). The aim of this study was to characterize the camel milk system supplying Nairobi city, and assess its governance, main challenges and the potential food safety risk practices using a value chain framework.

## 2 Material and methods

### 2.1 Scoping study for identification of the value chains to investigate

A cross-sectional study of the camel milk supply system serving Nairobi’s markets was carried out between August 2014 and January 2015. An initial scoping study was conducted through semi-structured interviews with officers from the Kenya Camel Association and the State Department of Livestock (Ministry of Agriculture, Livestock and Fisheries). During the interviews, participants were asked to create a preliminary mapping of the camel milk chains, through identification of the different supply chains, people involved in them and key locations (such as markets). As a result of this scoping work, the three most important chains supplying camel milk to Nairobi city were identified and visited for more detailed data collection. These were (1) the Isiolo value chain, (2) the Kajiado value chain, and (3) the Nanyuki value chain (processing company value chain).

Isiolo town (place of production for the Isiolo value chain) is located at about 300 km north of Nairobi. The town is characterised by both peri-urban and pastoral camel production systems and a thriving camel milk trade since the mid-1990s. This chain was identified as the main source of milk in the scoping study. Kajiado County (place of production of the Kajiado value chain) is located at about 150 km south of Nairobi. Since the late 2000s, the county has become an important centre of the camel milk trade, with increasing supply routes to Nairobi. Nanyuki town (place of production of the Nanyuki value chain, hereafter referred to as the processing company value chain) is located at about 200 km north of Nairobi. The chain was identified to be controlled and organized by one processing company, representing the most formal value chain. This was reported to be the only camel processing company supplying milk to Nairobi. The company was established in 2005 with the aim of processing camel milk sourced from Laikipia County and neighbouring Isiolo County.

Likewise, as result of the scoping study, Eastleigh market was identified as the hub of the camel milk trade in Nairobi city and therefore included in the study.

### 2.2 Data collection

Data collection was conducted across the three different value chains using the methodology described by Kaplisnky and Morris (2001) and as applied by Alarcon et al. (2017) and Carron et al. (2017). Authorisation to visit the various sites was provided by the Directorate of Veterinary Services and the County Directors of Veterinary Services in the respective study areas. With the help of various leaders in each market/chain node, the different stakeholders were identified and classified by their roles. Thereafter, for each type of stakeholder focus group discussions were organised. Discussions were conducted in the language of preference (English, Swahili or Somali) by the participants and with the use of translators.

Focus group discussions were carried out with the aim of collecting both qualitative and quantitative data on: i) structure of the chains, by showing both the origin and destination of the milk; ii) participants’ roles and their interactions with other stakeholders in the chains; iii) main rules and associations existing in the chains (informal, private standards and formal); iv) product differentiation characteristics and types of economic transactions involved; v) seasonal effects (on the milk supply, quality and distribution) ; vi) main challenges of the people working in the chains; and vii) waste management and food safety management practices. Data were collected by combining two methods: i) open ended questions by prompting participants to explain the various aspects of the value chains; and ii) creation of flowcharts with participants that indicate the flow of products, roles of people involved in the flows and the quantities of products traded. In addition, camel farms, milk bulking centres and markets were visited, and practices potentially risky for food safety identified.

To complement and validate the data obtained from the focus group discussions, semi-structured interviews with key informants were conducted. These were stakeholders whose position enabled them to have a wide and deep understanding the overall organisation and functionality of the camel milk value chain in Kenya. The aim of these interviews was to obtain additional qualitative and quantitative data on value chain aspects including: i) roles of stakeholders in each chain; ii) type and quantity of products in each chain; iii) overall governance in the different chains: in particular the role of government at both county and national level; and iv) waste management and food safety risks practices. Key informants included: a) veterinary officers, b) public health officers, c) livestock production officers, d) heads of different retailers’ associations, and e) non-governmental organisations within the camel milk subsector. In addition, the results from the focus group discussions were presented to these key informants to identify potential errors or misunderstanding of the results. In total eight focus group discussions and seven key informant interviews were conducted (Table 1).

**Table 1:** Summary of people interviewed during the study.

Place	Type of interview	Person	Number of people interviewed
Eastleigh	Focus group discussion	Isiolo milk traders	21
		Milk hawkers	7
		Kajiado milk traders	4
		Milk transporters	10
		Milk trader who knew the trading system	1
	Key informant	Representative of the milk traders	1
Isiolo	Focus group discussion	Isiolo milk traders	30
		Camel farmers	15
		Anolei camel milk bulking center manager	1
	Key informant	Sub-County Director of Public Health	1
		County Director of Veterinary Services	1
		County Director of Livestock Production	1
		Coordinator of Mobile pastoralism team	1
Nanyuki	Key informant	General manager of the milk processing factory	1
		Production manager	1
**Total**			**96**

All data from focus group discussions and key informant interviews were captured through digital audio recordings after signed consent from the participants was obtained.

### 2.3 Data analysis

By carefully listening of the audio recordings and reading of the written notes, data were collated in text documents, hereafter referred to as ‘templates’. These templates represented a first analysis stage, as data and major emerging themes were organised into sections: data regarding source and destination of milk. We performed a second stage of qualitative thematic content analysis by reading carefully all the templates. Emerging themes identified were then categorised as belonging in various high order nodes such as: a) informal or formal rules, b) associations, c) interaction with different stakeholders, d) challenges and e) food safety practices.

Following methods described in Taylor and Rushton (2011) and as applied by Alarcon et al. (2017), the value chain mapping was performed by drawing flow diagrams indicating and connecting the roles of people, type of products and the different places involved in the three identified chains. These flow diagrams were created by combining the different flowcharts obtained in each focus group discussion and interview; and by accounting for the emerging themes identified in the qualitative analysis stage. For analysis of chain governance the various emerging themes related to ‘interactions’; ‘rules and regulations’; ‘associations and dominance’; and ‘gender issues’ were used. Formal rules were defined as a set of rules, codes of conduct formalised and having a legislative backing; whereas informal rules were defined as overtly acknowledged rules and codes of conduct set by a group of persons to accomplish a particular goal without any legislative backing.

An association was defined as a group of individuals such as farmers, traders or transporters coming together for a common interest. Likewise for analysis of food safety, themes related to farms/milking/transport/market hygiene, training, waste management and animal health management were identified. Themes identified as related to challenges or barriers confronting the different stakeholders were grouped within nine major categories: (i) policy, (ii) marketing, (iii) financial, (iv) infrastructural, (v) relational, (vi) environmental, (vii) security, (viii) technological and ix) organisational. In addition, linkages between governance, challenges facing stakeholders and food safety – as reported by the stakeholders or interpreted by the main author – were identified.

## 3 Results

### 3.1 Value chain mapping

The Isiolo value chain accounted for 89% of milk brought to Nairobi city with a daily supply of 3000 L; Kajiado accounted for 5%, with a daily supply 120 L; and the milk processing company accounted for 6%, with a daily supply of 250 L of processed milk.

#### 3.1.1 Mapping of the Isiolo milk chain

Nomadic pastoralism, characterized by the irregular and constant movement of livestock in search of water and pastures, was identified as the main form of camel farming in this region. About 80% of pastoralists in this chain were classified as middle-scale with herd sizes between 50–100 camels.

**Fig. 1:**
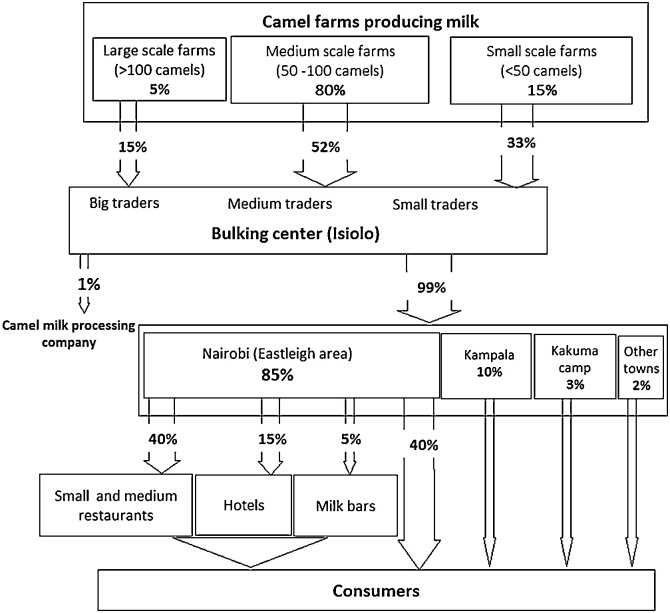
Isiolo Milk value chain profile: flowchart showing sources and retailing channels for milk. Percentages relate to the volume of milk traded. Of note, physical volume does not always equate to financial value.

Milk traders purchased milk from farmers, at their farms, and then transported it to a bulking centre in Isiolo town. At the bulking centre, the milk was weighed, tested for adulteration and bacterial levels, and kept in a cooling tank overnight, before being transported to Nairobi. Milk traders in Isiolo, who are predominantly women, reported that they were organised into a co-operative society. Three types of traders were identified: i) small traders supplying less than 50 L/day; ii) medium-sized traders supplying 50–100 L/day; and iii) large traders supplying more than 100 L per day. Medium traders represented the largest proportion of 52%, while small traders accounted for 33% and big traders 15%. Traders reported to transport the milk in 20 L plastic containers (not designed for milk transport), and using public service buses. In Nairobi’s Eastleigh market, milk traders received and distributed the milk to various places within the area, as well as to other parts of the city (Fig. 1). Traditionally, Eastleigh market was reported to be designated by the city council for selling imported clothes and other household wares, and there is no specific area for trading camel milk or other food items. Nonetheless, it is in this market where the camel milk trade was reported to occur. Traders stated that milk was sold directly to private consumers (for drinking and/or preparing tea), small and medium restaurants, three and four star hotels, and milk bars (defined as specialized retailers, often with an open front, or a counter where milk is sold).

Discussions with traders in Eastleigh revealed that 85% of the milk sold in this market and brought from Isiolo is consumed within the city, while 10% is exported to Kampala City, Uganda; 3% is sent to Kakuma refugee camp in Northern Kenya, and 2% is consumed in numerous local towns such as Nakuru, Eldoret, Kisumu and Mombasa.

#### 3.1.2 Mapping of the Kajiado milk chain

**Fig. 2:**
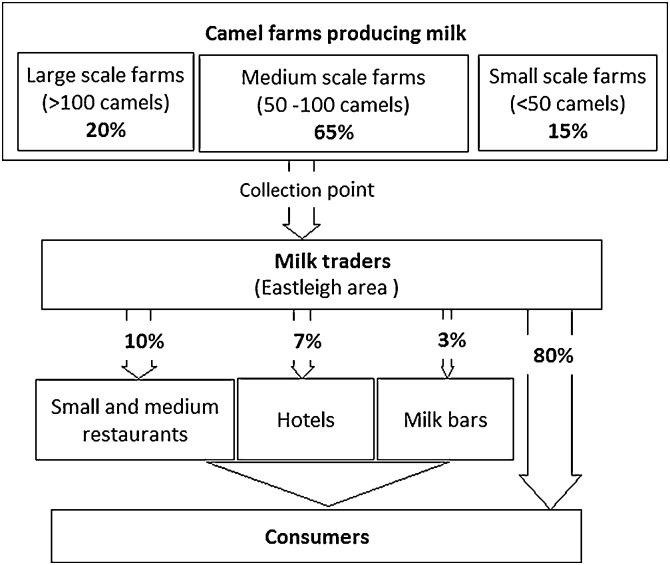
Kajiado milk value chain profile: the flowchart shows sources and retailing channels for milk. Percentages relate to the volume of milk traded. Of note, physical volume does not always equate to financial value.

For this chain, the majority (65%) of the camel herds were reported to be of medium-size (between 50–100 camels per farm). The proportion of large-scale pastoralists (20%) was slightly larger than in Isiolo (Fig. 2). Milk traders reported that they purchased milk from farmers at various camel farms and transported it, using off-road vehicles and motorbikes, to collection centres in Bisil and Kajiado towns. The milk was then transported in bulk to Eastleigh market, confirming the importance of this hub location.

In Eastleigh market, milk traders, who are predominantly men, received the milk and thereafter distributed it to different places within the area. Traders reported that 80% of the Kajiado milk in the Eastleigh market was sold to private customers for consumption (mostly in raw form) and/or for making tea; 10% to small and medium restaurants; 7% to large hotels and 3% to milk bars. As opposed to the Isiolo milk chain, here no milk was exported to other countries or transported to areas outside Nairobi.

#### 3.1.3 Mapping the processing company value chain

**Fig. 3:**
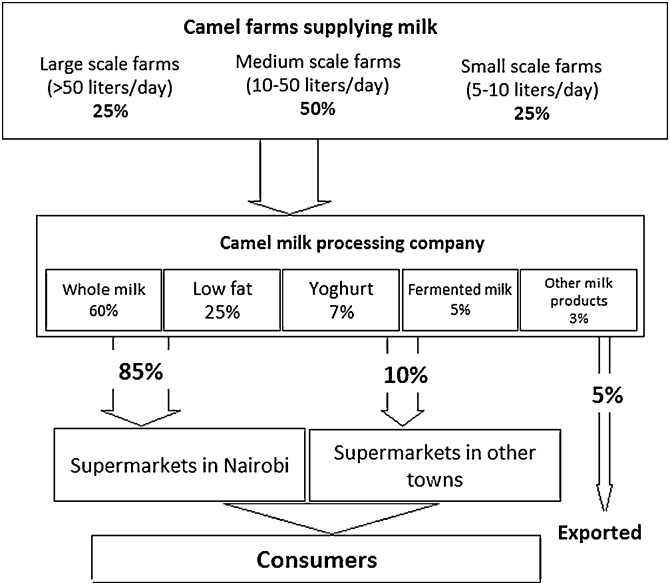
Camel milk processing company profile: the flowchart shows sources and retailing channels for milk and milk products. Percentages relate to the volume of milk traded. Of note, physical volume does not always equate to financial value.

Unlike the Isiolo and Kajiado milk chains, ranching was the main form of camel keeping in this chain. In this farming system camels are grazed in open grasslands and herded back in the evening into enclosed structures. Additionally, this farming system is characterised by a higher quality of veterinary care and management practices. Half of the total number of farms (50%) supplying milk in this chain were reported to produce 50 L of milk per day. The processing company reported that it worked directly with farmers, without relying on traders. Whole milk (pasteurized) was reported as the main processed product, comprising 60% of the total processed milk, while 25% was low fat boiled milk, and 7% yoghurt. Fermented milk and other milk products made up the remaining 5% (Fig. 3). The company estimated that approximately 85% of the processed camel milk products were sold to Nairobi city, mostly in large and medium sized supermarket outlets. On the other hand, 10% of the processed products were sold to other towns in Kenya, and 5% exported to regional and international markets.

#### 3.1.4 Seasonality

Stakeholders in the three chains reported that milk production and supply was dependent on rainfall patterns. March to May and October to December are the rainy seasons in the camel rearing areas and represent peak milk production and supply; while mid-July to September and January to February are the dry periods and represent a reduction in both milk production and supply. Participants in the three chains reported that despite seasonal fluctuations in the supply, the demand of milk was stable throughout the year. Hence, milk prices were reported to vary across seasons, peaking during the dry season, and dropping during the rainy season. Analysis of data from the focus group discussions, and key informant interviews, indicated that daily milk supply to Nairobi during the rainy season from Isiolo, Nanyuki and Kajiado increased to 5000 litres, 250 litres and 200 litres from the year average of 3000 litres, 150 litres and 120 L respectively.

### 3.2 Food safety risky practices reported along the value chains

#### 3.2.1 Farm level

Milking and milk handling hygiene measures were described as poor, particularly in the Isiolo and Kajiado chains. Specifically, the identified concerns were: (i) use of plastic milking cans (not designed for milk transport) that are difficult to clean, (ii) lack of hand washing during milking, and (iii) dirty milking environments. Farmers reported lack of adherence (mostly occasioned by low levels of awareness) to stipulated withdrawal periods for milk from camels undergoing antibiotic treatment. Milk traders, the processing company and public health officers identified the “smoking of milk” – a common cultural practice involving fumigation of milk containers with the burned woods of specific herbs – especially in the Isiolo milk chain as a potential risky practice. Farmers reported that the smoke was meant to prolong the shelf life of the milk and impart a distinct and, according to consumers, a desirable flavour to the milk.

#### 3.2.2 Bulking and transport level

A major concern for most of the stakeholders, particularly in Isiolo and Kajiado chains, was the lack of cold storage and refrigeration facilities along the chains (with the exception of cool tanks in the bulking centres). In Isiolo, traders used public service buses to transport milk to Nairobi. Likewise, all stakeholders, in particular government officials, reported that the use of plastic containers in milk handling and transportation was as a major concern, due to the difficulty to clean these efficiently.

#### 3.2.3 Retail level

In the Eastleigh market, milk traders operated along main roads due to a lack of a designated area for trading, therefore increasing the risk of milk contamination from a range of environmental sources. All interviewed stakeholders reported that most of the camel milk consumers did not boil camel milk before consumption – a serious risk practice to public health with respect to exposure to milk borne pathogens. Government officers estimated that 85% of the informally traded milk was consumed raw. The lack of adequate traceability system in Kajiado and Isiolo milk chains was reported as an important concern for food safety. Traders in both the Isiolo and Kajiado chains reported to pour spoiled milk down the sewer drains adjacent to their trading areas. This was common during the rainy season when milk supply was high.

Different stakeholders reported low levels of training on food safety, with the vast majority of farmers and traders relying on informal knowledge gained from other people in their social networks.

### 3.3 Governance themes and their linkages to food safety management

A summary of key governance themes and their linkages to food safety management is outlined in Table 2; they are further explained in the narrative below.

**Table 2:** A graphic representation of the framework used and summary of key governance themes and challenges, and how they relate with food safety management.

Themes			Linkage to food safety risk management
Governance	Stakeholders interactions	No formal contracts, except in the processing company value chain.	Food safety criteria not formally imposed in transactions, but only through informal agreements. Hence, this depends purely on individual stakeholders’ attitude, knowledge and awareness.
		Lack of involvement of government bodies in the Isiolo and Kajiado chains.	Lack of enforcement of food safety regulations and lack of surveillance on food safety risks.
		Non-Governmental Organisations (NGOs) play important role in support of Isiolo and Kajiado camel milk chains.	Food safety training is mainly dependent on NGOs actions.
	Rules and regulations	No formal rules in Isiolo and Kajiado chains.	Food safety depends on trust and religion. The latter as a sign of honesty for good (safe) business practice.
		Processing company has clear food safety private standards and government enforcement.	Capacity to implement effective food safety risk management.
		Lack of traceability	Considering the large number of stakeholders and general lack of formal organization, traceability was not a feature in the camel milk chains supplying Nairobi.
	Associations and dominance	Isiolo: Formal milk trader association that imposed informal rules and enforce them through sanctions	Traders represent the dominant group ability to implement effectively potential food safety management rules.
		Kajiado: No association along the value chain	No dominant group is able to establish compliance of food safety. Hence, this depends purely on individual relationships.
Challenges and Opportunities	Lack of specific policy framework for the camel milk trade		Inability to effectively regulate and enforce the food safety practices in the chains
	Lack of infrastructure - lack of good roads, cold chain and designation for market areas		Increase time for transport of milk to Nairobi without using cold chain.
	Lack of infrastructure - lack of designation of market areas for camel milk trade		Lack of effective food safety regulations. Increase exposure of milk to environmental food safety hazards.
	Financial and marketing challenges		Lack of capacity to invest in food safety equipment and training

#### 3.3.1 Stakeholders interactions

There was regular interaction between people within the described chains, yet there appeared to be little, if any, formalisation of contracts between them. The system was described as largely dynamic and ‘understood’ by all the different stakeholders.

In the Eastleigh market, traders from Isiolo and Kajiado chains had different trading areas and targeted different consumer groups. The majority of the stakeholders interviewed mentioned that government bodies, such the Kenya Dairy Board, had minimal roles, especially in the Kajiado and Isiolo milk chains. This indicates a potential gap of enforcement of foods safety legislation in these chains. However, the processing company chain revealed that government regulation was pronounced and influential in their operations. Various non-governmental organisations (NGOs), including international NGOs, were reported to be playing a significant role in trying to improve milk safety and reducing post-harvest losses in the Isiolo chain. Such activities included organising training on food safety for key stakeholders such as milk traders, transporters and farmers.

#### 3.3.2 Main rules and regulations

In the Isiolo and Kajiado chains stakeholders described a reliance on informal rules, such as those for setting a common price for the buying and selling of milk. Additionally, stakeholders in the Isiolo and Kajiado chains reported that informal rules (stipulated by the stakeholders themselves) were enforced and adhered to, with the risk of sanctions (imposed by the trader association) cited as the main reason for compliance. Sanctions mentioned included fines, suspension and/or expulsion from the trade. However, the processing company value chain was tightly coordinated and vertically integrated by the processor with clear set of formal rules, including government regulations and private standards.

In the informal milk chains, mutual trust, risk of sanctions and religion played an important role in preventing bad business practices, such as dishonesty in payments, and importantly in preventing some food safety risky practices such as milk adulteration. On the other hand, combination of government regulations and private standards in the processing company chain were highlighted as a major contributor to food safety. These contrasting characteristics of the two kinds of value chains relate to different food safety risky practices.

#### 3.3.3 Associations and dominance

In the Isiolo value chain, milk traders both in Nairobi and Isiolo itself had a formal association which facilitated the link with other stakeholders, particularly farmers and transporters. Through this association the traders had established a milk collection and a cooling centre in Isiolo and gained access to credit facilities. Farmers and milk transporters lacked such an association, despite their awareness of the importance of having one. The Isiolo milk traders therefore assumed the dominant role in the value chains supplying milk to Nairobi, owing to the greater volumes of milk traded by them. This dominance expresses itself in their ability to determine pricing, implement sanctions and the control of market information flow. Also, the milk traders were identified as the group with the potential to effectively impose food safety standards.

For the Kajiado value chain, stakeholders did not have any association and were not aware of the need for one. Hence, no structure to impose food safety norms, and this was dependent on individual relationships between stakeholders. By influencing the behaviour of other stakeholder in the value chains, dominant stakeholders and associations were identified as vital in preventing some food safety risky practices.

#### 3.3.4 Gender distribution

For the Isiolo milk chain, women were seen to control the trade both in Isiolo and Nairobi, with men assuming supporting roles, such as milk transport. Conversely, in the Kajiado milk chain, men dominated the chain with women providing supporting roles, such as hawking of milk and cleaning of milk containers. Akin to dairy milk chains, the processing company chain was largely dominated by men with women providing supporting roles.

### 3.4 Challenges and their linkages to food safety management

A summary of challenges affecting the value chain (and the stakeholders) and their linkages to food safety management is outlined in Table 2; they are further explained in the narrative below.

Policy and infrastructural challenges were identified as the main issues affecting 25% and 23% of stakeholders respectively. Lack of a specific policy framework governing the camel milk trade was cited as a significant hindrance especially in regulating food safety practices in the chains. Similarly, poor infrastructural investments such as: (i) inaccessible or unmaintained roads in the production areas, (ii) the lack of cold chain and (iii) designated camel milk market areas in Nairobi, were cited as major challenges. Infrastructural challenges were identified by all interviewed stakeholders as major hindrances to food safety. For instance, the lack of cold chain (a critical control point of microbial contamination) in all three chains was identified as a major hindrance to food safety. Similarly, lack of a designated area for trading camel milk unable the capacity for effective regulation of milk trade and increase the risk of milk exposure to environmental hazards. Financial and marketing challenges were identified by 18% of the stakeholders. High operational costs, low market for camel milk and a lack of access to credit facilities were identified as important challenges. This may represent a lack of capacity of stakeholders to invest in food safety equipment and training. About 20% of the farmers identified environmental challenges, such as diminishing land sizes and increasing droughts, as important to them.

## 4 Discussion

Three important value chains supplying camel milk to Nairobi city, including how governance themes and main challenges relate to food safety management, were identified and described. Mapping of the three value chains, in the largely unregulated camel subsector, helped in understanding the structure and complexity of the milk supply system. These results are consistent with (Musinga et al., 2008) who showed the importance of the various value chains supplying camel milk to consumers in Nairobi. Within the city, Eastleigh area was identified as the main market for camel milk. Eastleigh area is predominantly inhabited by members of the Somali community who are traditional consumers of camel milk, thereby providing a ready market (Lindley, 2007). Notwithstanding the influx of people of Somali origin, the demand of camel milk had markedly increased over the last five years primarily due to the perceived medicinal properties and associated health benefits of camel milk. This rapid increase in demand for camel milk may require a more organised supply system that is more closely regulated, in order to avoid risks related to pathogen transmission. Findings from this study show that unprocessed camel milk was sold onwards from the Nairobi trading hub to various towns and also exported internationally, mostly using public service vehicles; highlighting how future policies and research can help optimise issues related to food safety.

A range of stakeholders working in the three value chains were identified. Traders in the Kajiado and Isiolo chains played a pivotal role in the sourcing and supplying of milk to Nairobi. Various authors have highlighted the role of traders in the informal camel milk chain, both in Kenya and other countries (Nori et al., 2006; Seifu, 2007; Anderson et al., 2012; Wayua et al., 2013). Similarly, traders have a major role in the cattle milk trade in Kenya and other developing nations (Blackmore et al., 2015). To ensure sustainability and to strengthen these camel milk chains, policy makers may need to specifically consider the role of each stakeholder, with a focus on the dominant traders, while also considering other key groups along the food chains.

Most of the camel milk supplying Nairobi was informally traded, predominantly hawked (defined as selling or offering for sale of milk in public places), with minimal government involvement and no processing. Processed camel milk was available in only a select few high-end supermarkets. This is similar to what has been reported in other countries, such as Saudi Arabia, where the camel milk sector is dominated by the informal trade: not only in volume but also in the numbers of the stakeholders involved (Faye et al., 2014). Consumer preferences for unprocessed milk (mostly for cultural reasons), and low level awareness of camel milk amongst non-traditional consumers, are limiting factors in the wider expansion of this trade (Musinga et al., 2008).

Similar to previous studies, results from this study suggest that the camel milk subsector was largely unregulated with minimal government involvement (Anderson et al., 2010; Mwaura et al., 2015). This situation was particularly evident in the Isiolo and Kajiado milk chains. Informal trade presents a governance and regulatory dilemma to policy makers. On the one hand, whilst increases in regulatory involvement would potentially contribute to improvements in milk safety, and potentially opening up more markets for milk export, the current system is adapted to operate in a no regulation environment. It is uncertain to what extent the introduction of strict regulations may endanger the capacity of the informal chains to operate. Research on the impact of different regulation scenarios, their acceptability and efficacy in protecting stakeholders’ livelihood, maintain women empowerment (in particular in Isiolo chain) and protect public health is required. Considering the complexity of the camel milk trade in Kenya and the vital role played by the informal trade, understanding its dynamics and evaluating cases where policies and mechanisms have worked for stakeholders could guide the improvement of the informal trade in order to transition to more sustainable value chains. Drawing lessons from the Kenyan fresh fruit and vegetable industry value chain that demonstrates how informal arrangements can flourish in highly standardized value chains (Vorley et al., 2016), policy makers in the camel milk subsector could look beyond regulation to include initiatives that include equitable linkages between the informal and formal economies while ensuring inclusivity.

The Kajiado and Isiolo chains were managed with informal rules and there was a general absence of the enforcement of rules and regulations by relevant government bodies. In these chains, dominant traders and other stakeholders served as *de facto* rule enforcers, imposing rules and regulations upon other stakeholders through differential pricing and limiting information flow. These information asymmetries could be a source of major food safety management inefficiencies, and therefore it will be important to develop an understanding of whether the current institutional environment is adequate to manage food safety risks. Whilst reliance on informal regulation may lead to disadvantages for some of the stakeholders such as limited information flow, 'community governance' is often capable of enforcing norms; thereforeenhancing chain functionality and strengthening the contribution of formal regulations. Results from this framework analysis showed that Isiolo milk traders played a pivotal role in the governance of the camel milk chains supplying Nairobi. Policy makers aiming to make and implement policies in the milk chains may need to consider critical stakeholders such as the Isiolo milk traders while including the other stakeholders.

Other than Isiolo traders, stakeholders lacked a formal trade association, despite highlighting the importance of having one. Several studies have highlighted the pivotal role of associations in bringing together stakeholders and products, defining behaviours and perception towards value chains, and importantly achieving increased incomes for the various stakeholders, for instance, through improved market access or cost sharing (Thiele et al., 2011; Cavatassi et al., 2009). Whilst the Isiolo milk traders association provided such a platform, it lacked a coherent framework for proper governance and wider upgrading; thus failing to maximise the potential to have full market exploitation. Reiterating the importance of associations, value chain stakeholders (and policy makers) in the camel milk system could benefit from establishing and/or expanding the existing associations to create linkages amongst themselves.

Using a value chain approach this study highlights the significant role of women in the organisation and decision-making along the camel milk chains. Even in male-dominated chains such as Kajiado milk chain, women would contribute with some activities (such as hawking milk) vital to the chain’s functionality. Traditionally, in the dairy value chains, the role of women is concentrated at the production level with men taking major roles in decision making and value chain governance (Herrero et al., 2013). Future policies should consider the effect of commercialisation of the camel milk chains to avoid erosion of women’s control of the trade.

Challenges highlighted in this study were consistent with Musinga et al. (2008), who highlighted the lack of policy, financial and infrastructural challenges as the major constraints affecting the stakeholder. Results from this study illustrate the importance of performing a value chain analysis to identify the range of action points and upgrading strategies which, in combination, could significantly lead to improvements in traders’ incomes. For instance, by raising milk quality through hygienic milking and handling practices, and reducing post-harvest losses through the use of insulated milk storage and transportation, the stakeholders in the Isiolo and Kajiado milk chains can successfully improve milk outputs. Ultimately such initiatives will translate into increased incomes and improved food safety.

This study identifies the main risky practices likely to be present in the camel milk chains (from production to consumption) supplying Nairobi. The encountered risky practices may in part be a reflection of the interaction of multiple stakeholders separated by vast distances, high cost of compliance, and low enforcement capacity across the camel milk chains. Given the informal nature of these camel milk chains, simple innovations such as food grade containers and personal hygiene during milking and marketing can result in substantial improvements to food safety and quality. The observation that most of the traded milk was consumed raw is attributed to a long standing cultural belief that camel milk has curative abilities against jaundice, malaria and other diseases. A study in Ethiopia, for example, revealed that 100% of all camel milk consumers interviewed consumed raw milk (Seifu, 2007). Interestingly, a high level of trust was reported as a reason for low levels of adulteration in Kenya’s camel milk trade; unlike in the dairy cattle milk trade. These behavioural patterns highlight how aspects of food safety are embedded in broader sociocultural determinants, and more importantly how they may positively or negatively affect food safety (Blas and Kurup, 2010). Taken together, these results highlight the need for policy makers to consider motivational drivers behind human behaviour when designing risk management strategies, and to ensure that new policies are sensitive to existing sociocultural practices. By analysing the linkages between value chain governance, challenges and food safety this study highlights the role of a holistic view and understanding of potential risks practices safety when designing future policies and research aimed at enhancing management of food safety risks.

Occurrence of zoonotic and foodborne hazards that can be linked to camel milk has been documented in various studies (both in Kenya and other camel keeping regions), including brucellosis (Shimol et al., 2012; Garcell et al., 2016), antimicrobial resistance (Fazlani et al., 2011), and salmonellosis (Kaindi et al., 2011). Future research could benefit from investigating the microbiological hazards along the identified camel milk value chains and their importance (in terms of severity of microbial contamination), with particular emphasis on the identified “risk” points.

The finding that the majority of the stakeholders lacked adequate formal training in food safety is in agreement with, and supported by, previous studies of informal food systems (Roesel and Grace, 2014). The only formal training offered was mostly dependent on NGOs activity. Therefore, and importantly, to keep these chains healthy, stakeholders (including consumers) and policy makers could consider initiatives that support regular and appropriate education on safe food handling.

The combination of increasing preference for camels as a climate adaptation strategy (Watson et al., 2016) and increased demand for camel milk due to the associated health benefits has led to increased camel production in traditional and non-traditional camel keeping communities globally. As reported in this study, this shift to peri-urban camel production system has been described as an emerging trend in numerous places (Correra et al., 2008; Faye et al., 2012). Taken together, whilst findings from this study are particularly relevant to Kenya, the approach can easily be applied in other countries.

This study had several limitations. First, the results represent participants’ perceptions on the structure and functionality of the camel milk value chains. Second, this study investigated the major camel milk value chains, as identified through the scoping study, thus other minor chains were not investigated. However, the importance of other chains in the supply of camel milk to Nairobi is believed to be minimal. Finally, lack of quantitative data was a limitation: for some chains, actual data on supply quantities was not available and estimations from the stakeholders present in the interview had to be made. The impact of these ‘educated guesses’ was minimised by triangulating and validating the data with interviews of different key informants that have an overall understanding of the markets, such as public health officers and livestock production officers.

## 5 Conclusion and recommendations

In conclusion, this study provides an overall understanding of the camel milk value chains supplying Nairobi and their food safety risk management. Using a value chain mapping framework, three major value chains supplying camel milk to Nairobi city were identified: the Isiolo, Kajiado and camel milk processing company chains. Mapping of people and products highlighted the kinds of products traded, sources and destination of the products, role played by the different stakeholder and importantly the governance structure in these chains. This framework can be used by policy makers firstly to better the camel milk system structure and functionality, but potentially also to identify opportunities for upgrades, and in particular in relation to food safety management and control.

Future intervention policies are needed to empower value chain stakeholders to (i) improve food safety measures, (ii) ensure financial inclusivity, and (iii) promote overall chain growth. Taking the long view, to achieve inclusivity and sustainability of the camel milk chains supplying Nairobi while making them safer; instead of taking a “formalising the informal trade” approach, regulatory involvement should consider a combination of mechanisms that complement each other.

## Ethical approval

Ethical approval for this study was obtained from the ILRI Institutional Research Ethics Committee (ILRI IREC) (project reference: ILRI-IREC2015/01).

## Conflict of interest

The authors have no conflicts of interest to declare.
